# A Rare Case of Carpenter Syndrome and Its Unique Association With Chronic Kidney Disease

**DOI:** 10.7759/cureus.62823

**Published:** 2024-06-21

**Authors:** Pranjal Kashiv, Shubham Dubey, Sunny Malde, Sushrut Gupta, Twinkle Pawar, Kapil N Sejpal, Prasad Gurjar, Amol Bhawane, Priyanka Tolani, Charulata P Bawankule, Amit Pasari, Manish Balwani

**Affiliations:** 1 Nephrology, Jawaharlal Nehru Medical College, Datta Meghe Institute of Medical Sciences, Wardha, IND; 2 Medicine, Jawaharlal Nehru Medical College, Datta Meghe Institute of Medical Sciences, Wardha, IND; 3 Nephrology, All India Institute of Medical Sciences, Nagpur, IND; 4 Internal Medicine, Jawaharlal Nehru Medical College, Datta Meghe Institute of Medical Sciences, Wardha, IND; 5 Nephrology, Saraswati Kidney Care Center, Nagpur, IND; 6 Nephrology, Saraswati Kidney Care Centre, Nagpur, IND

**Keywords:** genetic pathways, syndromic interplay, rab23 mutations, chronic kidney disease, carpenter syndrome

## Abstract

Carpenter syndrome, characterized by RAB23 mutations, is a rare autosomal recessive disorder distinguished by unique features such as craniofacial anomalies, congenital heart disease, brachydactyly, and obesity. This syndrome's rarity, with an estimated prevalence of one in a million births, poses diagnostic challenges due to its diverse clinical spectrum. Notably, this case report highlights an unusual association of Carpenter syndrome with chronic kidney disease (CKD), underscoring the need for further exploration into the syndromic interplay and shared genetic pathways. The distinctive manifestation of CKD in the context of Carpenter syndrome adds a novel dimension, emphasizing the importance of timely diagnosis and comprehensive care. Further research is warranted to unravel the intricate genetic and molecular pathways underlying the syndrome's diverse manifestations, shedding light on potential shared mechanisms and paving the way for targeted interventions and enhanced patient care.

## Introduction

Carpenter syndrome, or acrocephalopolysyndactyly type II, is a rare autosomal recessive disorder associated with biallelic mutations in RAB23. This guanosine triphosphate hydrolase (GTPase) on chromosome 6 is a crucial negative regulator of the sonic hedgehog signalling pathway. Carpenter syndrome presents with various manifestations, including congenital heart diseases, developmental disorders, mental retardation, obesity, hypogonadism, thyroid abnormalities, umbilical hernia, bone anomalies, and frequent respiratory infections [1,2]. Distinctive features of Carpenter syndrome involve brachydactyly, syndactyly of hands and feet, short stature, and obesity. Cardiovascular anomalies are common, such as ventricular or atrial septal defects, patent ductus arteriosus, pulmonic stenosis, tetralogy of Fallot, or transposition of the great arteries. The prevalence is approximately one in a million births [3,4]. Unlike the classic presentation, this case report focuses on a variant of Carpenter syndrome associated with chronic kidney disease (CKD). Notably, this case, likely the first reported in India, underscores the need to explore the potential connections between Carpenter syndrome and CKD. The case report aims to share insights into this uncommon syndromic variant and its link to CKD.

## Case presentation

We present a compelling case of an 18-year-old female referred to us for elevated creatinine levels, initially admitted to a local hospital for severe breathlessness, swelling in both legs and facial puffiness. On evaluation, she was found to have acute decompensated heart failure with cardiogenic shock. A 2D echocardiography depicted dilated cardiomyopathy with severe left ventricular dysfunction having an ejection fraction of 20%, mild mitral regurgitation, mild tricuspid regurgitation, dilated left ventricle, and global left ventricle (LV) hypokinesia. Routine investigations indicated urea levels of 60 mg/dl, creatinine levels of 3.6 mg/dl, proteinuria of 3+, and urine pus cells at 20-25 cells/hpf, with a urine protein creatinine ratio (UPCR) of 1.75. (Table 1)

**Table 1:** Laboratory parameters and investigations of the patient

Investigation	Result/Findings	Reference range
Urea levels	60 mg/dl	19-43 mg/dl
Creatinine levels	3.6 mg/dl	0.66-1.25 mg/dl
Proteinuria	3+	Less than 1+
Urine pus cells	20-25 cells/hpf	1-2 cells/hpf
Urine protein creatinine ratio (UPCR)	1.75	<0.15

**Figure 1:**
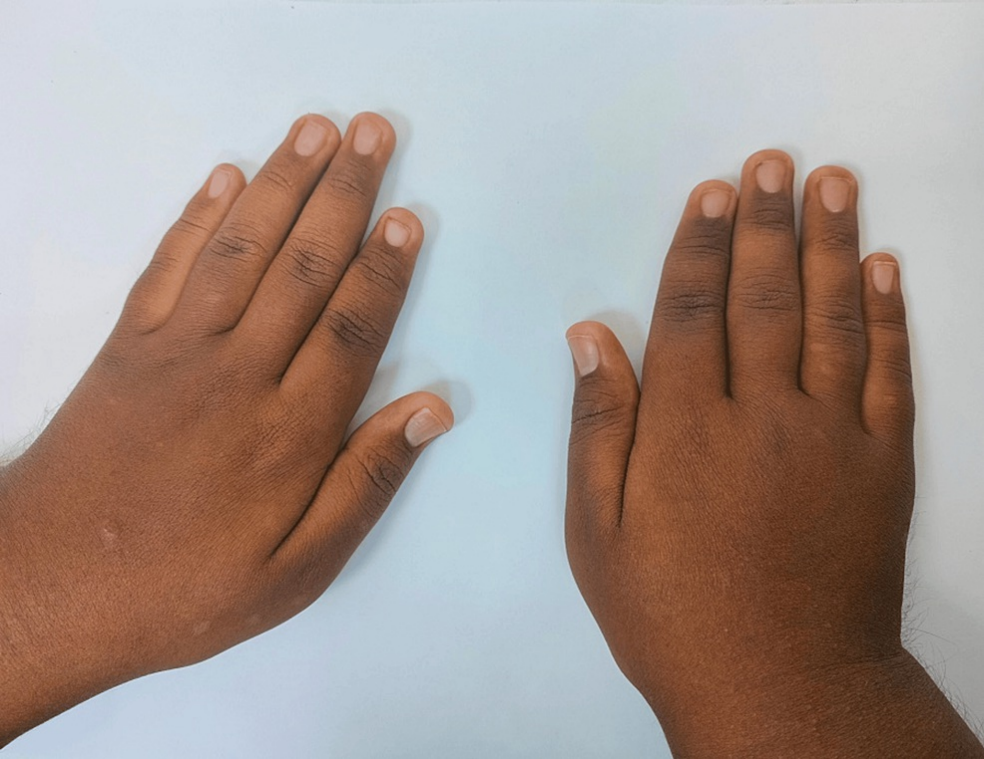
Hands with brachydactyly

Further examination revealed atrophic left kidney and right kidney showing raised echotexture with loss of corticomedullary differentiation, as well as additional findings of truncal obesity, brachydactyly (Figure 1), hypothyroidism, and craniofacial abnormalities.

Acute-on-CKD was identified with asymptomatic pyuria responding to antibiotics, resulting in a decrease in creatinine levels to 2.2 mg/dl. Upon obtaining a detailed history from her parents, it was revealed that she had exhibited bulging of the forehead, right leg syndactyly (involving the first and second toes), and finger deformities since birth. Parents took the child to a pediatrician who, on detailed examination, found to have features of scaphocephaly with trigonocephaly, low-set ears, hypotelorism, micrognathia, short neck, brachydactyly, dysmorphic facies, congenital heart disease (atrial septal defect), and craniofacial dysostosis. An X-ray of the skull revealed fused sagittal and metopic sutures, resulting in a narrow skull. Coronal and lambdoid sutures were smaller than normal, indicating premature fusion of the sagittal suture and altered skull shape. The diagnosis was a dolichocephalic skull with sagittal suture synostosis, and a morcellation procedure was performed at three months of age. 

Throughout the pregnancy, no complications were reported, and the mother had no history of radiographic examination, drug ingestion or any abdominal trauma. The infant, born to a 32-year-old father and 24-year-old mother, marked the second live birth with no family history of congenital anomalies. Upon evaluation, both parents and the older brother were normal. Detailed history and examination raised suspicion of a genetic disorder, leading to a genetic evaluation that revealed a homozygous mutation (RAB23 712 T>G). The diagnosis of Carpenter syndrome, also known as acrocephalopolysyndactyly type 2, a craniosynostosis syndrome, was confirmed. This unique case involves CKD coexisting with Carpenter syndrome. The patient is currently under follow-up, maintaining stable creatinine levels of around 2-3 mg/dl; her ejection fraction is now 40%, and she held adequate urine output.

## Discussion

The historical exploration of Carpenter syndrome, an autosomal recessive disorder first identified by George Carpenter in 1901, has unfolded gradually over several decades. Initially categorized among the acrocephalopolysyndactyly syndromes, this rare condition exhibits a distinctive array of features, including brachydactyly, acrocephaly, and syndactyly in the hands, along with toe abnormalities such as syndactyly and preaxial polydactyly [5]. Our case report introduces a unique dimension by documenting Carpenter syndrome concurrent with CKD. The clinical spectrum of Carpenter syndrome encompasses craniosynostosis, facial dysmorphism, and various limb anomalies, presenting challenges in both diagnosis and management [6]. Our patient's clinical and radiological features align with the established profile of Carpenter syndrome, emphasizing the syndrome's characteristic facial abnormalities and distinctive hand and foot anomalies.

Radiological scrutiny further confirmed the Carpenter syndrome diagnosis, and genetic analysis revealed a homozygous mutation RAB23 712 T>G in the RAB23 gene. The significance of this finding lies in the role of RAB23 as a critical negative regulator of the sonic hedgehog signalling pathway, underscoring the genetic underpinnings of Carpenter syndrome. This aligns with the broader understanding of Carpenter syndrome's genetic basis, as highlighted by Jenkins et al.'s comprehensive study, elucidating the gene's location on chromosome 6p12.1-q12 and reporting multiple genetic mutations [2]. Introducing CKD into the Carpenter syndrome narrative raises intriguing questions about the syndromic interplay and shared genetic pathways. The elusive pathogenesis of CKD in Carpenter syndrome beckons further exploration, necessitating a deeper investigation into the specific mechanisms connecting RAB23 mutations to CKD.

Timely genetic analysis is crucial for informed interventions and improved prognostic outcomes. This case underscores the critical importance of prompt diagnosis and treatment. Pediatricians and physicians must be vigilant, recognizing the unique link between Carpenter syndrome and CKD. This complex association emphasizes that healthcare professionals must stay informed and consider such interconnections in their practice, reinforcing the significance of early genetic identification for optimal patient care. Given the unique intersection of Carpenter syndrome with CKD presented in this case, there is a compelling need for further research in this area to unravel the intricate genetic and molecular pathways that underlie the syndrome's diverse manifestations, shedding light on potential shared mechanisms and paving the way for targeted interventions and enhanced patient care.

## Conclusions

Carpenter syndrome, with its complex clinical presentation and genetic underpinnings, is notably associated with chronic kidney disease. Our case illustrates the diverse symptoms linked to this syndrome, from craniosynostosis to obesity and cardiac issues. The discovery of a homozygous mutation in the RAB23 gene underscores its role in Carpenter syndrome and the sonic hedgehog signaling pathway. This highlights the need for vigilance among physicians, particularly pediatricians and geneticists, in identifying potential associations with CKD. Timely diagnosis and comprehensive management are crucial, as demonstrated by our case, which marks the first documented instance of Carpenter syndrome coinciding with CKD in India. These findings emphasize the importance of understanding the varied clinical presentations of Carpenter syndrome and advocating for further research into its genetic mechanisms and complexities.
